# Probabilistic Damage Detection of a Steel Truss Bridge Model by Optimally Designed Bayesian Neural Network

**DOI:** 10.3390/s18103371

**Published:** 2018-10-09

**Authors:** Tao Yin, Hong-ping Zhu

**Affiliations:** 1 School of Civil Engineering, Wuhan University, Wuhan 430072, China; 2 School of Civil Engineering & Mechanics, Huazhong University of Science and Technology, Wuhan 430074, China; hpzhu@mail.hust.edu.cn

**Keywords:** structural health monitoring, probabilistic damage detection, truss bridge, model class selection, Bayesian neural network

## Abstract

Excellent pattern matching capability makes artificial neural networks (ANNs) a very promising approach for vibration-based structural health monitoring (SHM). The proper design of the network architecture with the suitable complexity is vital to the ANN-based structural damage detection. In addition to the number of hidden neurons, the type of transfer function used in the hidden layer cannot be neglected for the ANN design. Neural network learning can be further presented in the framework of Bayesian statistics, but the issues of selection for the hidden layer transfer function with respect to the Bayesian neural network has not yet been reported in the literature. In addition, most of the research works in the literature for addressing the predictive distribution of neural network output is only for a single target variable, while multiple target variables are rarely involved. In the present paper, for the purpose of probabilistic structural damage detection, Bayesian neural networks with multiple target variables are optimally designed, and the selection of the number of neurons, and the transfer function in the hidden layer, are carried out simultaneously to achieve a neural network architecture with suitable complexity. Furthermore, the nonlinear network function can be approximately linear by assuming the posterior distribution of network parameters is a sufficiently narrow Gaussian, and then the input-dependent covariance matrix of the predictive distribution of network output can be obtained with the Gaussian assumption for the situation of multiple target variables. Structural damage detection is conducted for a steel truss bridge model to verify the proposed method through a set of numerical case studies.

## 1. Introduction

Recent structural damage or component failure has raised public awareness of the need for improved infrastructure safety and maintenance. It is of great practical significance to regularly evaluate and assess the service status of civil infrastructure. There has been great interest in the development of structural health monitoring (SHM) methodology, based on vibration measurements in recent decades [1]. Structural damage generally leads to the reduction of local stiffness, which has an influence on the dynamic characteristic parameters, and changes of these parameters before and after damage are further utilized by the vibration-based structural damage detection methods to identify the damage location and related extent. Different methods have been developed in various aspects of this area so far, such as dynamic analysis methods [2,3,4], probabilistic methods [5,6,7,8,9,10], model reduction-based methods [11,12,13], artificial intelligence methods [14,15,16], modal identification methods [17,18,19,20], wavelet-based methods [21,22], optimal sensor configurations [23,24,25], and signal processing techniques [26], et al.

Among these methods, artificial neural networks (ANNs) have not been specifically developed for structural damage detection, but their pattern matching capability makes them very promising to be employed as a tool for this purpose. It is obvious that the complexity of the ANN models, including the number of hidden layers and the number of hidden neurons, impacts significantly on the training process of the ANN, as well as the performance of the trained ANN, especially for simulating a complicated functional relationship. If these numbers are too small, the obtained ANN might not be able to capture the true behavior of the training data. In contrast, if they are too large, the trained ANN may produce outputs that will fluctuate in the region between training data points. Thus, proper design of the structure of ANN models, with suitable complexity, is necessary to ensure the successful application of ANN-based structural damage detection methods. However, the ANN model is usually determined by experience or rule of thumb only, and very limited research works exist in the area of ANN-based damage detection, addressing the issue of designing ANN models properly [27,28,29]. Other than the number of hidden neurons, the type of transfer (activation) function utilized in the hidden layer is also a non-negligible factor with respect to the ANN design, since the nonlinearity of the transfer function has an effect on the generalization ability of neural networks of the trained ANN.

In the traditional ANN approach as mentioned above, the values of the network parameters, including weights and biases, are estimated from the training data set by minimizing the sum of squared errors, representing the error between the target variables and the output of the neural network. More importantly, neural network learning can also be presented in the framework of Bayesian inference. Starting from the early research works related to Bayesian neural network [30,31], there has been a growing interest in the application of Bayesian inference theory in the field of neural networks [32,33,34,35,36,37]. By noting the significance of the ANN design as mentioned previously, the selection of the number of neurons in the hidden layer for the Bayesian neural network has been addressed [28]; however, to the best of our knowledge, the issues of selection of the transfer function, with respect to the Bayesian neural network, has not yet been addressed in previous studies. In addition, one of the significant features of the Bayesian neural network is the capability of quantifying the error or uncertainty of the network output, by employing the Bayesian inference approach. Nevertheless, most of the research activities in the literature for addressing the predictive distribution of neural network output only deal with a single target variable [38,39,40], whereas the distribution over network output for multiple target variables is seldom mentioned.

In this paper, the Bayesian neural networks with multiple target variables is optimally designed for the purpose of probabilistic structural damage detection; additionally, the simultaneous selection of numbers of neurons and the corresponding transfer function in the hidden layer to obtain a neural network with suitable complexity is well addressed. Furthermore, by assuming that the posterior distribution of network parameters is approximated as a sufficiently narrow Gaussian, so that the nonlinear network function is approximately linear with respect to the network parameters over the region of parameter space, the input-dependent covariance matrix of predictive distribution, being approximated also as a Gaussian, can be obtained for the case of multiple target variables. The feasibility and validity of the proposed method is verified by the numerical case studies of a steel truss bridge model.

## 2. Theoretical Development

The multi-layer feedforward neural network is commonly employed for ANN-based structural damage detection in the literature [41], and it has been proved to be capable of approximating any functional relationship between inputs and outputs with only one hidden layer [42]. In the present paper, without loss of generality, the proposed method focuses on the design of the structure of the hidden layer for a single-hidden-layer feedforward Bayesian neural network, since a linear function is always utilized as the transfer function in the output layer. This thus involves the selection of the number of neurons in the hidden layer, as well as the corresponding transfer function of the neurons in the same layer. The Bayesian neural network investigated in this paper updates the weight and bias values according to Levenberg–Marquardt optimization routine through the Bayesian regularization procedure by minimizing a linear combination of squared errors and weights.

The set of model classes for the Bayesian neural network in the present paper are defined as $M_{1},M_{2},\cdots ,M_{N_{M}}$, representing the network model with the different number of neurons and various types of transfer functions in the hidden layer, and $N_{M}$ is the total number of model classes considered. Consider the problem of predicting multiple target variables $t\in R^{N_{O}\times 1}$ from a vector $x\in R^{N_{I}\times 1}$ of inputs by utilizing the Bayesian neural network approach. $N_{I}$ and $N_{O}$ are the number of neurons in the input and output layers. Let $D_{N}=\left\{\left\{x_{1},t_{1}\right\},\left\{x_{2},t_{2}\right\},\cdots ,\left\{x_{N},t_{N}\right\}\right\}$ denote the input–output training data set which is generated from the finite-element analysis of target structure, and N is the total number of training data sets. For the structural damage detection problem investigated in this paper, the changes of natural frequencies and normalized partial mode shapes corresponding to the measured degrees of freedom (DOF) for the first few modes before and after damage are utilized as pattern features for the damage, which correspond to the ANN input. The damaged members that are to be identified relate to the ANN output. Thus, the aim of the Bayesian neural network design is to select the most plausible class of models by using data $D_{N}$ from $N_{M}$ prescribed classes of Bayesian neural network models to approximate the functional relationship defined by the input and output data set.

It is assumed that for the jth class of model $M_{j}$, that the conditional distribution $p(t|x,w_{j};M_{j})$ is Gaussian with an x-dependent mean given by the output of a neural network model $y\left(x,\;w_{j};M_{j}\right)$. Also, the multiple target variables t are assumed to be independent conditional on the inputs x and network parameters $w_{j}$ including weights and biases with shared noise precision parameter $\beta_{j}$, which represents the precision of the Gaussian noise. Then, the conditional distribution of the target values with respect to the jth class of models is given by [38]:

(1) $$p(t|x,\;w_{j},\beta_{j},M_{j})=N\left(t|y\left(x,\;w_{j};M_{j}\right),\;\beta_{j}^{-1}I_{N_{O}}\right)$$

where $I_{N_{O}}$ is an identity matrix in dimension $N_{O}$.

Given the input−output training data set $D_{N}$, one can construct the corresponding likelihood function as:

(2) $$\begin{array}{cc} p\left(D_{N}|w_{j},\beta_{j},M_{j}\right) & =\prod_{n=1}^{N}N\left(t_{n}|y\left(x_{n},\;w_{j};M_{j}\right),\;\beta_{j}^{-1}I_{N_{O}}\right)=\\ & ={\left(\frac{\beta_{j}}{2\pi }\right)}^{NN_{o}/2}\exp\left(-\frac{\beta_{j}}{2}\sum_{n=1}^{N}{\left\|y\left(x_{n},\;w_{j};M_{j}\right)-t_{n}\right\|}^{2}\right) \end{array}$$

where ‖·‖ represents the Euclidean norm.

Similarly, a prior distribution can be chosen to be Gaussian over the uncertain network parameters $w_{j}$ as the following:

(3) $$P\left(w_{j}|\alpha_{j},M_{j}\right)=N\left(w_{j}|0,\;\alpha_{j}^{-1}I_{W_{j}}\right)={\left(\frac{\alpha_{j}}{2\pi }\right)}^{W_{j}/2}\exp\left(-\frac{\alpha_{j}}{2}{\left\|w_{j}\right\|}^{2}\right)$$

where $I_{W_{j}}$ is a $W_{j}\times W_{j}$ identity matrix. $W_{j}=N_{I}+N_{O}+N_{H}$ is the dimension of the weight vector, and $N_{H}$ is the number of neurons in the hidden layer.

By following the Bayes’ theorem, the posterior distribution of the network parameters $w_{j}$ for the model class $M_{j}$ is given by:

(4) $$p\left(w_{j}|D_{N},\alpha_{j},\beta_{j},M_{j}\right)=\frac{p\left(D_{N}|w_{j},\beta_{j},M_{j}\right)p\left(w_{j}|\alpha_{j},M_{j}\right)}{p\left(D_{N}|\alpha_{j},\beta_{j},M_{j}\right)}$$

which, however, is non-Gaussian due to the consequence of the nonlinear dependence of the network function $y\left(x,\;w_{j};M_{j}\right)$ on the network parameters $w_{j}$.

By utilizing the Laplace approximation, one can seek a Gaussian approximation to the posterior distribution $p\left(w_{j}|D_{N},\alpha_{j},\beta_{j},M_{j}\right)$ in Equation (4) at a (local) maximum of the posterior, say $w_{j,\mathrm{MP}}$, which can be obtained through the standard nonlinear optimization routine. As usual, it is convenient to maximize the logarithm of the posterior, which can be written as:

(5) $$\ln\left[p\left(w_{j}|D_{N},\alpha_{j},\beta_{j},M_{j}\right)\right]\propto -\frac{\beta_{j}}{2}\overset{N}{\underset{n=1}{\sum }}{\left\|y\left(x_{n},\;w_{j};M_{j}\right)-t_{n}\right\|}^{2}-\frac{\alpha_{j}}{2}{\left\|w_{j}\right\|}^{2}$$

which corresponds to a regularized sum-of-squares error function, while the two hyperparameters $\alpha_{j}$ and $\beta_{j}$ are fixed and known at this moment.

Then, from Equation (5), a local Gaussian approximation can be built in by evaluating the matrix of second derivatives of the negative log posterior distribution at the maximum of the posterior, and it is given by:

(6) $$p\left(w_{j}|D_{N},\alpha_{j},\beta_{j},M_{j}\right)=N\left(w_{j}|w_{j,\mathrm{MP}},\;H_{j}^{-1}\left(w_{j,\mathrm{MP}}\right)\right)$$

where $H_{j}\left(w_{j,\mathrm{MP}}\right)={-\nabla \nabla \ln\left[p\left(w_{j}|D_{N},\alpha_{j},\beta_{j},M_{j}\right)\right]|}_{w_{j}=w_{j,\mathrm{MP}}}$ is the Hessian matrix evaluated at $w_{j,\mathrm{MP}}$.

By making use of the evidence framework, together with the Gaussian approximation to the posterior utilizing the Laplace approximation, the marginal likelihood or evidence for the two hyperparameters is obtained by integrating the network parameters as:

(7) $$\begin{array}{cc} p\left(D_{N}|\alpha_{j},\beta_{j},M_{j}\right) & =\int^{\text{​}}p\left(D_{N}|w_{j},\beta_{j},M_{j}\right)p\left(w_{j}|\alpha_{j},M_{j}\right)dw_{j}\\ & ≃p\left(D_{N}|w_{j,\mathrm{MP}},\beta_{j},M_{j}\right)p\left(w_{j,\mathrm{MP}}|\alpha_{j},M_{j}\right){\left(2\pi \right)}^{W_{j}/2}{\left|H_{j}\left(w_{j,\mathrm{MP}}\right)\right|}^{-1/2} \end{array}$$

In the evidence framework, the point estimates for hyperparameters $\alpha_{j}$ and $\beta_{j}$ can be obtained by maximizing $\ln\left[p\left(D_{N}|\alpha_{j},\beta_{j},M_{j}\right)\right]$ with respect to $\alpha_{j}$ and $\beta_{j}$ at the maximum of the posterior $w_{j,\mathrm{MP}}$ [38], respectively, as:

(8) $$\frac{1}{\alpha_{j,\mathrm{MP}}}=\frac{1}{\gamma_{j,\mathrm{MP}}}{\left\|w_{j,\mathrm{MP}}\right\|}^{2},\;\;\frac{1}{\beta_{j,\mathrm{MP}}}=\frac{1}{NN_{O}-\gamma_{j,\mathrm{MP}}}\overset{N}{\underset{n=1}{\sum }}{\left\|y\left(x_{n},\;w_{j,\mathrm{MP}};M_{j}\right)-t_{n}\right\|}^{2}$$

where $\gamma_{j,\mathrm{MP}}=W_{j}-\alpha_{j,\mathrm{MP}}\mathrm{tr}\left(H_{j}^{-1}\left(w_{j,\mathrm{MP}}\right)\right)$ represents the effective number of parameters corresponding to the jth model class. Equation (8) presents a practical iterative procedure for estimating the hyperparameters during the training process of Bayesian neural network.

To select the most plausible model class among $N_{M}$ prescribed model classes for the Bayesian neural network, the probability of a model class $M_{j}$ conditional on the given set of input-target training data $D_{N}$ should be calculated. This can be obtained by following the Bayes’ theorem as:

(9) $$p\left(M_{j}|D_{N}\right)=\frac{p\left(D_{N}|M_{j}\right)\pi \left(M_{j}\right)}{\sum_{j=1}^{N_{c}}p\left(D_{N}|M_{j}\right)\pi \left(M_{j}\right)}$$

where the prior probability $\pi \left(M_{j}\right)$ on the model class $M_{j}$, for j=1 to $N_{M}$, satisfies $\overset{N_{c}}{\underset{j=1}{\sum }}\pi \left(M_{j}\right)=1$. As there is generally no prior information about each class of models for the purpose of structural damage detection, it is simply assumed hereafter that each individual model class possesses the same initial plausibility, i.e., $\pi \left(M_{j}\right)=1/N_{M}$. The factor $p\left(D_{N}|M_{j}\right)$ is the most important term in Equation (9), and it is known as the evidence for the model class $M_{j}$ giving the set of input–output training data $D_{N}$. Generally, the class of models to be used is the one that maximizes the posterior probability $p\left(M_{j}|D_{N}\right)$, or equivalently, maximizes the model evidence $p\left(D_{N}|M_{j}\right)$ with respect to $M_{j}$.

It is noted that instead of utilizing the usual Bayesian treatment for hyperparameters involving marginalization over all possible values, the model evidence $p\left(D_{N}|M_{j}\right)$ presented in Equation (9) can be approximated by substituting the values of hyperparameters $\alpha_{j,\mathrm{MP}}$ and $\beta_{j,\mathrm{MP}}$ obtained at the maximum of the posterior $w_{j,\mathrm{MP}}$ from the iterative optimization procedure given in Equation (8) into the marginal likelihood in Equation (7), i.e.,

(10) $$p\left(D_{N}|M_{j}\right)≃p\left(D_{N}|w_{j,\mathrm{MP}},\beta_{j,\mathrm{MP}},M_{j}\right)p\left(w_{j,\mathrm{MP}}|\alpha_{j,\mathrm{MP}},M_{j}\right){\left(2\pi \right)}^{W_{j}/2}{\left|H_{j}\left(w_{j,\mathrm{MP}}\right)\right|}^{-1/2}$$

It is important to note that the form of the evidence $p\left(D_{N}|M_{j}\right)$ given in Equation (10) is consistent with that given by the authors of [43], that is, the evidence consists of two terms, $p\left(D_{N}|w_{j,\mathrm{MP}},\beta_{j,\mathrm{MP}},M_{j}\right)$ and $p\left(w_{j,\mathrm{MP}}|\alpha_{j,\mathrm{MP}},M_{j}\right){\left(2\pi \right)}^{W_{j}/2}{\left|H_{j}\left(w_{j,\mathrm{MP}}\right)\right|}^{-1/2}$, namely, the likelihood factor and the Ockham factor. Specifically, the likelihood factor favors more complex model classes. Thus, it will be higher for those model classes making the probability of the data $D_{N}$ higher, implying a better fit to the data. The Ockham factor, however, imposes a penalty against the complexity of the specified model class. The balance between these two factors allows one to select the most probable model class through a mathematically rigorous and robust way, which is just complex enough to fit the given data. In this study, the class of models to be selected is the one possessing the highest value of evidence, i.e., maximum posterior probability, among the entire set of model classes for the given set of train data. It is also noted that because the corresponding numerical values are usually very large, the logarithmic form of the evidence is taken in order to avoid the computational problem during the procedure of model class selection. This yields that:

(11) $$\begin{array}{c} \ln\left[p\left(D_{N}|M_{j}\right)\right]≃\frac{NN_{O}}{2}\ln\beta_{j,\mathrm{MP}}-\frac{\beta_{j,\mathrm{MP}}}{2}\sum_{n=1}^{N}{\left\|y\left(x_{n},\;w_{j,\mathrm{MP}};M_{j}\right)-t_{n}\right\|}^{2}\\ +\frac{W_{j}}{2}\ln\alpha_{j,\mathrm{MP}}-\frac{\alpha_{j,\mathrm{MP}}}{2}{\left\|w_{j,\mathrm{MP}}\right\|}^{2}+\frac{W_{j}}{2}\ln\left(2\pi \right)-\frac{1}{2}\ln\left|H_{j}\left(w_{j,\mathrm{MP}}\right)\right| \end{array}$$

where the sum of the terms in the first row gives the logarithm of the likelihood factor, and that in the second row represents the logarithmic expression of the Ockham factor.

In addition, it should be noted that if Equation (9) is applied for identifying the ‘optimal’ class of Bayesian neural network models by direct comparison of the conditional probability $p\left(M_{j}|D_{N}\right)$, the total number of model classes $N_{M}$ to be considered in the identification process should be specified at the beginning. If $N_{M}$ is too small, the ‘optimal’ model class might not be included in the study. However, if this number is too large, the computational consumption would be unaffordable. Thus, instead of directly comparing the all $N_{M}$ model classes and picking up the ‘best’ one, the number of neurons as well as the transfer function in the hidden layer is identified by following a computationally efficient algorithm [29]. Denote $N_{T}$ as the number of transfer functions involved for comparison, and the main procedure of the algorithm is summarized as following:

1. Initialize the index of the type of hidden layer transfer function i=1 (outer loop).
2. Initialize the index of number of hidden neurons k=1 (inner loop), and calculate the corresponding log evidence $\ln\left[p\left(D_{N}|M_{j\left(i,k\right)}\right)\right]$ in Equation (11), where j depends on both i and k.
3. Increase the index k by 1, and calculate the log evidences $\ln\left[p\left(D_{N}|M_{j\left(i,k+1\right)}\right)\right]$, which is compared with $\ln\left[p\left(D_{N}|M_{j\left(i,k\right)}\right)\right]$; If $\ln\left[p\left(D_{N}|M_{j\left(i,k+1\right)}\right)\right]<\ln\left[p\left(D_{N}|M_{j\left(i,k\right)}\right)\right]$, then $M_{j\left(i,k\right)}$ is the model class with ‘optimal’ number of hidden neurons as for the ith transfer function, and the inner loop will stop; Or, the algorithm will increase the index k by 1 and repeat this step; Record the log evidence $\ln\left[p\left(D_{N}|M_{j\left(i,k_{i}\right)}\right)\right]$ related to the ‘optimal’ number of hidden neurons for the present transfer function.
4. Increase the index of transfer function i by 1, repeat steps 2 and 3, and record the log evidence $\ln\left[p\left(D_{N}|M_{j\left(i+1,k_{i+1}\right)}\right)\right]$ related to the ‘optimal’ number of hidden neurons for the (i+1)th transfer function; Compare $\ln\left[p\left(D_{N}|M_{j\left(i+1,k_{i+1}\right)}\right)\right]$ with $\ln\left[p\left(D_{N}|M_{j\left(i,k_{i}\right)}\right)\right]$, and record the larger one.
5. If $i<N_{T}$, set i=i+1, and repeat step 4; Otherwise, if $i=N_{T}$, the whole algorithm will stop, and output the ‘optimal’ number of hidden neurons $k_{i^{*}}$ as well as the related hidden layer transfer function index $i^{*}$ corresponding to the ‘optimal’ model class $M_{j\left(i^{*},k_{i^{*}}\right)}$ with recorded largest log evidence $\ln\left[p\left(D_{N}|M_{j\left(i^{*},k_{i^{*}}\right)}\right)\right]$.

On the other hand, it is assumed that the identified most plausible model class through the above design procedure is $M_{K}$, and $K=j\left(i^{*},k_{i^{*}}\right)$. Then, the posterior of the network parameters given in Equation (6) can be employed to produce a distribution over the network outputs, and the predictive distribution is obtained by marginalizing with respect to this posterior distribution, given as:

(12) $$p(t|x,D_{N},M_{K})=\int^{\text{​}}p(t|x,\;w_{K},\beta_{K,\mathrm{MP}},M_{K})p\left(w_{K}|D_{N},\alpha_{K,\mathrm{MP}},\beta_{K,\mathrm{MP}},M_{K}\right)dw_{K}$$

It is noted that this integration is analytically intractable due to the nonlinearity of the network function $y\left(x,\;w_{K};M_{K}\right)$ as a function of the network parameters $w_{K}$. Under such circumstances, an approximation to evaluate this integral is generally required. To make progress, it is assumed that the covariance of posterior distribution of network parameters is small, so that the network function is approximately linear with respect to the parameters over the region of parameter space for which the posterior probability is significantly nonzero [38]. By making a Taylor series expansion of the network function around the maximum of the posterior $w_{K,\mathrm{MP}}$ and retaining only the linear terms, the conditional distribution of the target values can be written as:

(13) $$p\left(t|x,\;w_{K},\beta_{K,\mathrm{MP}},M_{K}\right)≃N\left(t|y\left(x,\;w_{K,\mathrm{MP}};M_{K}\right)+J_{K}\left(x,w_{K,\mathrm{MP}}\right)\left(w_{K}-w_{K,\mathrm{MP}}\right),\;\beta_{K,\mathrm{MP}}^{-1}I_{N_{O}}\right)$$

where the mean is a linear function of $w_{K}$, and $J_{K}\left(x,w_{K,\mathrm{MP}}\right)={\nabla y\left(x,\;w_{K};M_{K}\right)|}_{w_{K}=w_{K,\mathrm{MP}}}$ is the **x**-dependent Jacobian matrix of the vector-valued network function evaluated at the maximum of the posterior $w_{K,\mathrm{MP}}$, and can be calculated by utilizing the finite difference approach.

Then, by noticing that the posterior distribution of weights is approximated as a sufficiently narrow Gaussian, one arrives at a Gaussian distribution over the outputs of the network as the following:

(14) $$p(t|x,D_{N},M_{K})≃N\left(t|y\left(x,\;w_{K,\mathrm{MP}};M_{K}\right),\;\Sigma_{K}\left(x,w_{K,\mathrm{MP}}\right)\right)$$

where the predictive distribution is approximated as a multivariate Gaussian, the mean of which is given by the network function $y\left(x,\;w_{K,\mathrm{MP}};M_{K}\right)$ with the network model parameters set to their maximum of the posterior -estimate. The corresponding **x**-dependent covariance matrix is given by:

(15) $$\Sigma_{K}\left(x,w_{K,\mathrm{MP}}\right)=\beta_{K,\mathrm{MP}}^{-1}I_{N_{O}}+J_{K}\left(x,w_{K,\mathrm{MP}}\right)H_{K}^{-1}\left(w_{K,\mathrm{MP}}\right)J_{K}^{T}\left(x,w_{K,\mathrm{MP}}\right)$$

It is apparent that the **x**-dependent covariance matrix $\Sigma_{K}\left(x,w_{K,\mathrm{MP}}\right)$ consists of two terms. The first one reflects the intrinsic noise on the target variable, whereas the second is an **x**-dependent term expressing uncertainty, due to the uncertainty of the weights.

## 3. Case Studies

**Figure 1:**
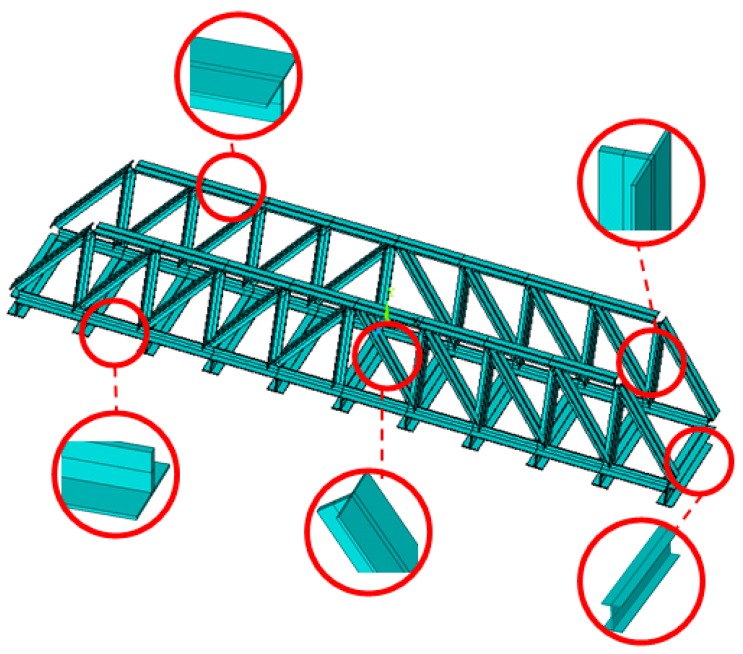
Steel truss bridge model.

**Figure 2:**
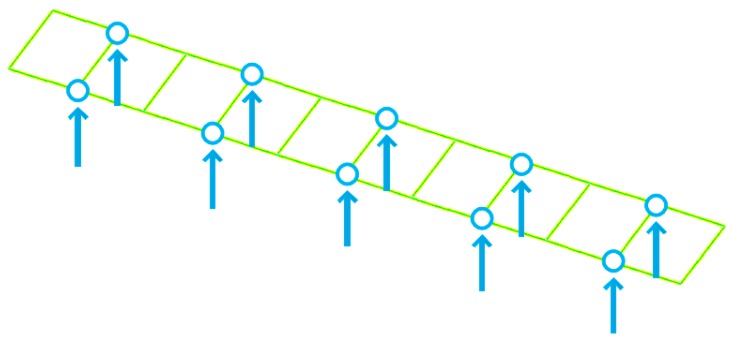
Configuration of ten measurement points on the bridge deck.

**Figure 3:**
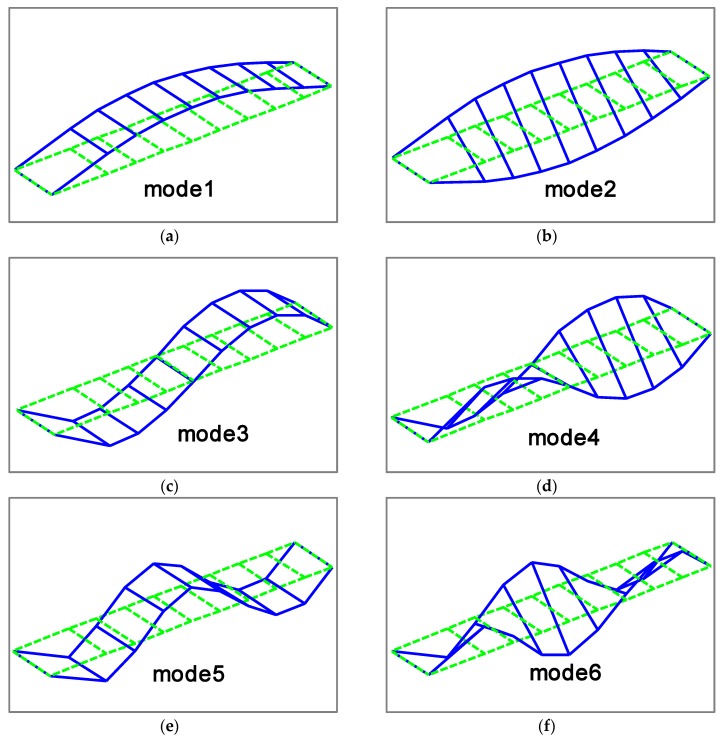
First six mode shapes: (**a**) Mode 1 (74.91 Hz); (**b**) Mode 2 (83.67 Hz); (**c**) Mode 3 (221.13 Hz); (**d**) Mode 4 (240.72 Hz); (**e**) Mode 5 (312.06 Hz); (**f**) Mode 6 (370.63 Hz).

To verify the proposed methodology, numerical case studies are conducted in this section for a simply-supported steel truss bridge model, as shown in Figure 1. The space steel truss bridge, including 11 crossbeams, has a total length of 2.8 m, a width of 0.48 m and a height of 0.4 m, respectively. Except for the cross-section of the crossbeams being the I-steel type, the other components, including the upper and lower chords, and the diagonal and vertical rods, are all made of a pair of angle irons with the same size. Sectional and material properties of the truss bridge model are shown in Table 1. Each component of the truss bridge model is discretized into one beam finite element, and the total number of such elements for the entire finite element model is 85. The configuration of measurement points on the bridge deck is shown in Figure 2, and the ten measurement points, evenly located on the both sides of bridge deck, measure the vertical motion of the bridge structure. The first six mode shapes of the steel truss bridge deck in intact status are shown in Figure 3, including the first three vertical vibration and torsional modes.

**Figure 4:**
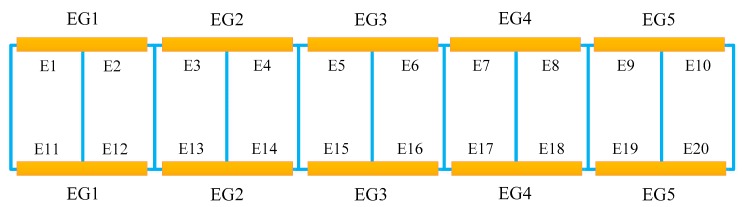
Configuration of element groups for generating training data.

The damage is considered on the lower chord of the steel truss bridge model by reducing the bending stiffness, which is achieved by reducing the Young’s Modulus of the potentially damaged structural member with a scaling factor in the present study, and there is a total of 20 potential damage locations, which are denoted by E1 to E20, as shown in Figure 4, respectively. However, the excessive number of damaged locations to be identified will result in too many output neurons, which is not conducive to Bayesian neural network training and subsequent damage identification procedures. Thus, one can consider combining these 20 possible damage locations into 5 groups, represented by EG1 to EG5, respectively, and each group consists of 4 lower chord elements, as shown in Figure 4 and Table 2. In this way, the number of output neurons in the neural network is greatly reduced, from 20 to 5. For example, the element group EG2 includes four individual elements E3, E4, E13, and E14. In such circumstances, if any one or more of the four elements in the same element group are damaged, the damage is only reflected by the overall bending stiffness reduction of the element group EG2. In addition, considering the fact that in the initial stage of the damage development, the number of simultaneously damaged elements would be very few, it is reasonably assumed herein that there are, at most, two damaged elements at the same time for the truss bridge model. Furthermore, three damage extents of 0, 20%, and 40% with respect to the element group, respectively, are utilized to generate the training data for the Bayesian neural network. It is also noted that for each sample of training data, the differences of the natural frequencies and mode shapes before and after the damage for the steel truss bridge model is stacked into a vector and used as the network input. It is noted that although all modes can be calculated from the finite element model, only modal parameters of the first two modes are utilized for damage identification of the steel truss bridge model, in order to evaluate the proposed method with limited information to mimic reality, and the total number of input neurons is 22.

**Figure 5:**
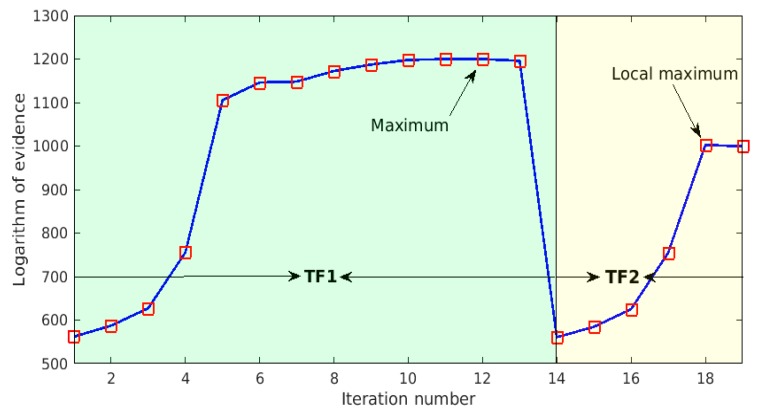
Iteration history of the model class selection procedure.

This example considers two commonly used types of hidden layer transfer functions, i.e., tansig and satlins [44], labeled as TF1 and TF2 respectively. Figure 5 shows the iterative curve of the proposed Bayesian neural network design procedure. The abscissa represents the number of iterations, while the ordinate denotes the logarithm of evidence. This curve can be clearly divided into two segments, related to the hidden layer transfer functions TF1 and TF2, respectively. It is clear from the results corresponding to TF1 that, as the number of neurons in the hidden layer increases, the log-evidence value gradually increases. Specifically, as shown in Figure 5, when the number of hidden neurons is less than five, the increase of log evidence along with the number of hidden neurons is particularly significant, whereas after that, this increase becomes very gentle. It can thus be seen that, the number of neurons in the hidden layer has a great influence on the performance of the Bayesian neural network, especially when the number of hidden neurons is small; however, as the number increases to a certain extent, the influence becomes less obvious. This clearly indicates that the number of neurons in the hidden layer cannot be too small; otherwise, the Bayesian neural network might not work well. As the number of hidden neurons increases to 12, the log-evidence value reaches the maximum value with respect to the hidden layer transfer function TF1, and the continuous increase of the number of hidden neurons results in a decrease in the log evidence. Thus, the network design algorithm switches to the next candidate transfer function TF2. Similarly, with the increase of number of hidden neurons, the log evidence increases significantly when the number of hidden neurons is relatively small, and gradually reaches its local maximum value. This, however, is smaller than the largest log-evidence value found previously for TF1, and the algorithm stops and finally determines TF1 as the best transfer function under consideration, and the corresponding optimal number of hidden layer neurons is found to be 12 at the same time.

**Figure 6:**
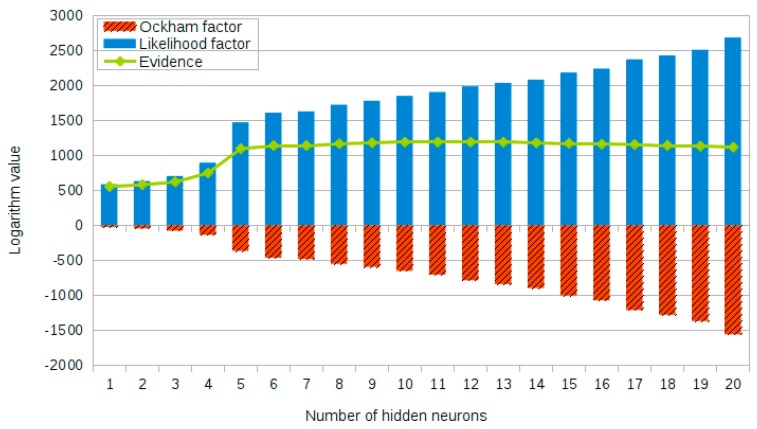
Logarithm value of likelihood factor, Ockham factor and evidence.

In order to interpret the principle of the proposed network design approach more clearly, taking the hidden layer transfer function TF1 as an example, Figure 6 shows the full picture of the log evidence, the likelihood factor, and the Ockham factor when the number of neurons in the hidden layer gradually increases from 1 to 20, with the number of input and output neurons remaining unchanged. It can clearly be seen from this figure that, as the number of neurons in the hidden layer gradually increases, the structure of the Bayesian neural network model becomes more complex, and network model is expected to better fit the training data, which is reflected by the increase of likelihood factor. At the same time, the absolute value of the Ockham factor gradually increases, which represents a gradual increase in penalty for the complexity of the network model, and the detailed numerical results are also presented in Table 3 for reference (the results corresponding to the ‘optimal’ numbers of hidden neurons for both TF1 and TF2 are shown in bold). Therefore, under the joint influence of these two factors, the Bayesian network model with the optimal number of hidden neural can be reasonably determined. In addition, it should be realized here that the network performance is sharply increased for a small number of hidden neurons, and then gradually decreased after the log evidence reaches its maximum value, but the magnitude of the decrease is not too obvious. This clearly indicates that the normal operation of the Bayesian neural network requires a sufficient number of hidden neurons, and it also shows that the network optimization design is particularly important in the case of fewer neurons in the hidden layer.

**Figure 7:**
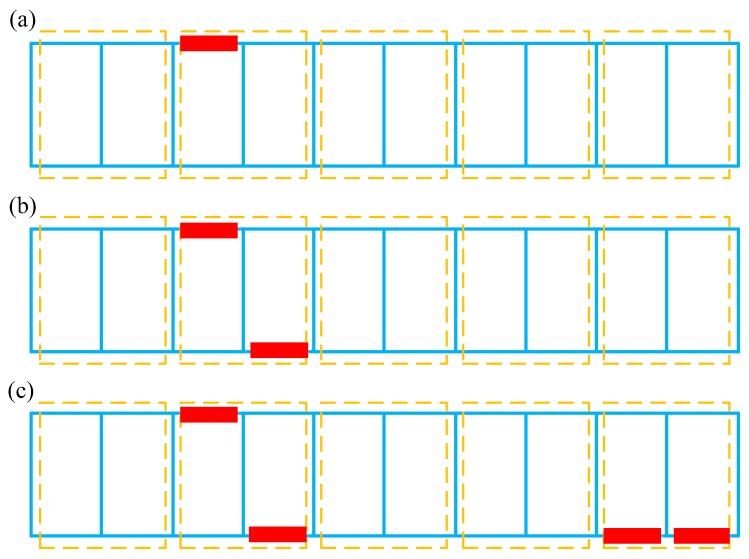
Damage configurations for the truss bridge model: (**a**) Case 1 (E3); (**b**) Case 2 (E3 and E14); (**c**) Case 3 (E3 and E14 and E19 and E20).

In this paper, three damage cases are considered for the steel truss bridge model. As shown in Figure 7 and Table 4, the damage is simulated by reducing bending stiffness of the corresponding individual element. Specifically, Case 1 considers the single damage situation, and the damage only occurs in element E3, which belongs to the 2nd element group EG2. Case 2 relates to a double-damage condition, and the damage exists simultaneously in E3 and E14. The last one is a multi-damage case, based on Case 2, where the simultaneous damage on E19 and E20 is further involved. Referring to the element group configuration as shown in Table 2 and Figure 4, both Case 1 and Case 2 represent damage that occurred in EG2, whereas the latter shows a relatively greater damage extent. At the same time, Case 3 assumes that EG2 and EG5 are simultaneously damaged. Without loss of generality, the modal parameters utilized for the training data of the neural network involve 1% Gaussian white noise, and the previously optimized Bayesian neural network is employed for the network training and subsequent probabilistic damage detection process. The prediction results of the network output are shown in Table 5. It can be seen from this table that the results of each damage case and the corresponding degree of uncertainty can be successfully identified. Comparing the damage identification results of Case 1 and Case 2, it is clearly seen that the uncertainty of identified damage is not much different under both cases, but the identified damage extent of EG2 for Case 2 is significantly larger than Case 1. This is consistent with the prescribed damage for each case as defined in Table 4, i.e., in Case 2, element group EG2 contains two damaged individual elements at the same time. For the last case, the proposed method also successfully identifies the actually damaged element groups EG2 and EG4, and the uncertainty associated with the damage identification results in such a case is also successfully quantified.

**Figure 8:**
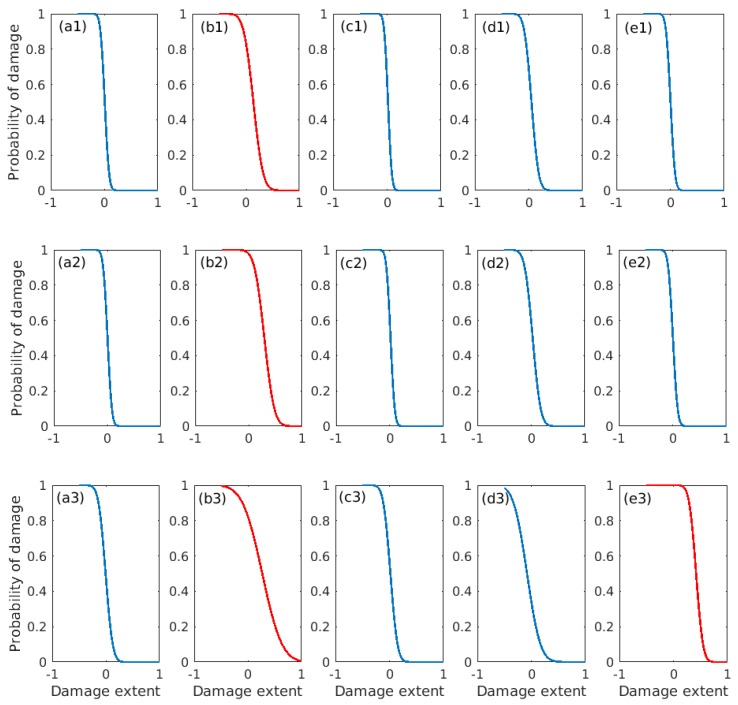
Probability of damage: (**a1**)−(**e1**) Case 1; (**a2**)−(**e2**) Case 2; (**a3**)−(**e3**) Case 3.

Additionally, the damage probabilities [45] for this truss bridge model are further quantified to seek a better interpretation of the damage detection results, the data of which are shown in Figure 8 for all three cases under consideration. It can be found for Case 1 that the 2nd element group of the truss bridge model has the greatest probability of damage, as indicated in Figure 8(a1−e1), since the corresponding probability value is somewhat more significant than others. But it is also realized that, as compared to the relatively small extent of damage in EG2, EG4 also possesses a certain degree of damage probability due to the symmetry of the steel truss bridge model. This might not be virtually revealed by directly inspecting the results presented in Table 5**,** and it will possibly cause some interference to the damage judgment for the present case. As for Case 2, there are also similar observations derived from Figure 8(a2–e2), i.e., EG2 is also the most likely damaged, as compared to the remaining element groups. The probability of damage in EG2 is much more significant than others, since it is a more serious damage configuration as compared to Case 1. For Case 3, it is very obvious from Figure 8(a3−e3) that both EG2 and EG5 have a relatively large possibility of damage, which coincides well with the actual damage configuration prescribed for this case.

## 4. Conclusions

For the purpose of probabilistic damage detection based on vibration measurements, the artificial neural network embedded with Bayesian inference for multiple target variables is optimally designed through simultaneous selection of the number of hidden neurons and the type of transfer functions in the hidden layer to obtain a network model with suitable complexity. The validity of the proposed methodology is verified by the numerical case studies conducted for a steel truss bridge model. The obtained results clearly show that under the joint influence of the likelihood and Ockham factors, the proposed network design procedure allows one to determine the optimal architecture of Bayesian neural network model, including a suitable hidden layer transfer function, as well as the optimal number of hidden neurons at the same time via a mathematically rigorous and robust way, which is just complex enough to fit the training data. In addition, it is also revealed that for the present example, regardless of the specific form of the hidden layer transfer function, the normal operation of the Bayesian neural network requires a sufficient number of neurons in the hidden layer, and the network optimization design is particularly important in the situation of fewer neurons in the hidden layer. Furthermore, in addition to the successful identification of damage locations and relative extents, the multivariate predictive distribution of the Bayesian neural network output derived in this paper can be efficiently utilized for quantifying the uncertainty associated with the statistical damage identification results. It should be pointed out that both the Hessian matrix of the logarithm of the posterior and the input-dependent Jacobian matrix of the vector-valued network function evaluated at the maximum of the posterior of weight vector, are calculated by the finite difference approach, which is very time consuming, especially for the weight vector with high dimension. Thus, one of the directions of future work will focus on reducing such computational cost for the purpose of practice application. Furthermore, only numerical examples are employed in the present paper, and the proposed method will be further validated by the experimental case studies in the coming publications.

## Figures and Tables

**Table 1:** Sectional and material properties of the steel truss bridge model.

Parameter Name	Values
Young’s modulus	2.06 × 10^11^ N/m^2^
Mass density	7.85 × 10^3^ kg/m^3^
Cross-sectional area (Angle iron)	0.47 × 10^−3^ m^2^
Cross-sectional area (I-steel)	1.57 × 10^−3^ m^2^
Moment of inertia (Angle iron)	0.11 × 10^−6^ m^4^
Moment of inertia (I-steel)	2.21 × 10^−6^ m^4^
Bridge span	2.8 m
Bridge width	0.48 m
Bridge height	0.4 m

**Table 2:** Definition of element groups for the steel truss bridge model.

Element Groups	Number of Elements	Included Elements
EG1	4	{E1, E2, E11, E12}
EG2	4	{E3, E4, E13, E14}
EG3	4	{E5, E6, E15, E16}
EG4	4	{E7, E8, E17, E18}
EG5	4	{E9, E10, E19, E20}

**Table 3:** The results of model class selection for the Bayesian neural network.

Transfer Functions	Number of Hidden Neurons *N_H_*	Logarithm of		
		Evidence	Likelihood Factor	Ockham Factor
TF1 (tansig)	8	1172.42	1724.16	−551.74
	9	1187.23	1780.93	−593.70
	10	1197.86	1849.99	−652.12
	11	1199.74	1905.59	−705.85
	**12**	**1199.93**	**1986.27**	**−786.34**
	13	1195.72	2037.02	−841.30
	14	1186.62	2083.90	−897.28
	15	1178.15	2183.16	−1005.01
TF2 (satlins)	1	560.74	588.01	−27.27
	2	584.43	633.46	−49.03
	3	625.17	705.89	−80.73
	4	754.87	895.72	−140.84
	**5**	**1002.49**	**1265.83**	**−263.34**
	6	999.30	1280.24	−280.94
	7	995.60	1290.60	−295.00
	8	993.11	1302.23	−309.12

**Table 4:** Damage cases considered for the steel truss bridge model.

Cases	Damaged Elements (Stiffness Reduction)	Element Groups
Case 1	E3 (30%)	EG2
Case 2	E3 (30%) & E14 (50%)	EG2
Case 3	E3 (30%) & E14 (50%) & E19 (30%) & E20 (50%)	EG2 & EG5

**Table 5:** Prediction results of the Bayesian neural network.

Network Output	Case 1		Case 2		Case 3	
	Identified	STD Value	Identified	STD Value	Identified	STD Value
***t*_1_** (EG1)	0.0095	0.06	0.0052	0.06	−0.0049	0.10
***t*_2_** (EG2)	**0.1408**	0.14	**0.2938**	0.15	**0.2689**	0.29
***t*_3_** (EG3)	0.0175	0.05	0.0161	0.06	0.0182	0.10
***t*_4_** (EG4)	0.0523	0.10	0.0287	0.12	−0.0812	0.20
***t*_5_** (EG5)	0.0047	0.06	0.0066	0.07	**0.4202**	0.10
